# Authors' Reply

**DOI:** 10.1371/journal.pmed.0020187

**Published:** 2005-06-28

**Authors:** Erick Turner, Martin Tramèr

**Affiliations:** **1**Portland VA Medical Center and Oregon Health and Science UniversityPortland, OregonUnited States of America; **2**Geneva University HospitalsGenevaSwitzerland

We appreciate Dr. Miller's contribution to this debate [1]. Whether one uses his phrase “established treatment” or “proven therapy,” we urge caution in using such a litmus test to decide whether the use of placebo is or is not acceptable. Such terms beg to be defined carefully. Should nonsteroidal anti-inflammatory drugs be considered “proven” for arthritis, despite their problems with assay sensitivity [2,3]? Dr. Miller states that there is no established treatment for adenomatous polyps. However, there is evidence from epidemiological studies and clinical trials supporting the use of aspirin and other nonsteroidal anti-inflammatory drugs for this condition [4–10]. Armed with such evidence—whether it qualifies aspirin as “proven therapy” is open to subjective interpretation—many placebo opponents, we maintain, would argue for an active-controlled design as a more ethical alternative to the placebo-controlled design actually used in APPROVe [11].

Dr. Miller focuses on the question of defending the use of placebo in the APPROVe study. This focus reflects the prevailing bias, which is to choose when it is acceptable to use placebo rather than to choose when it is acceptable to omit placebo. This bias is evident in the current version of the Declaration of Helsinki, with wording such as, “Extreme care must be taken in making use of placebo-controlled trials.” Thus, the use of placebo is typically presumed “guilty until proven innocent,” while active-controlled designs are presumed “innocent until proven guilty.” The declaration is silent on the possibility that omitting placebo can lead to problems, too, as we have now witnessed with Vioxx.

So perhaps the more important question should be whether it was defensible to exclude placebo in the VIGOR study. Dr. Miller acknowledges that placebo would have provided a better assessment of the safety signal in the VIGOR study. Indeed, because placebo was not used, the authors were able to plausibly conclude that the difference between the two groups was due to naproxen causing benefit rather than to Vioxx causing harm [12]. (The plausibility of this conclusion has since been questioned [13,14].) This misleading safety signal only delayed the withdrawal of Vioxx. Its design was superficially ethical, but science was not advanced, and the public health was ill-served.

One might protest that these comments are made with the benefit of hindsight. It is true that the medical community at large did not become aware of this safety issue until September 2004, when the results of the placebo-controlled APPROVe study were made public and Vioxx was withdrawn. However, according to David Graham's testimony to the United States Senate [15] and a report on internal Merck documents [16], there was good reason for concern about a possible safety signal before 1999, when Vioxx was approved and recruitment for VIGOR began [15].

If the VIGOR study had included a placebo arm, the truth about Vioxx could have been learned in February 2001 instead of September 2004 [14]. That is over 180 weeks during which the now infamous “two to four jumbo jetliners” were allowed to continue “dropping from the sky every week” [15]. Using the midpoints of Graham's range estimates, this works out to 94,500 excess heart attacks and strokes, including 33,000 deaths, in the US alone.

The authors of the VIGOR study said, “We could not include a placebo group” [12]. Was the idea of including a placebo arm suggested but rejected as “unethical,” even though rescue medication could have been used? Or did they take the path of least resistance in the interest of rapid institutional review board approval and ease of patient recruiting? Whatever the reason, the decision to omit placebo led to ambiguity and inaction.

In clinical trials, whether one looks at efficacy (please see our opening argument in this debate [17]) or safety, omitting placebo often muddies the scientific waters and places the public health at increased risk. Good science and good ethics cannot be divorced from one another. We believe these considerations should factor into discussions on the ethics of clinical trial design. Before we experience another Vioxx, we hope that a future version of the Declaration of Helsinki will add, “Extreme care must be taken when omitting placebo.”
